# *GLP1R* Gene Expression and Kidney Disease Progression

**DOI:** 10.1001/jamanetworkopen.2024.40286

**Published:** 2024-10-25

**Authors:** Jefferson L. Triozzi, Zhihong Yu, Ayush Giri, Hua-Chang Chen, Otis D. Wilson, Brian Ferolito, T. Alp Ikizler, Elvis A. Akwo, Cassianne Robinson-Cohen, John Michael Gaziano, Kelly Cho, Lawrence S. Phillips, Ran Tao, Alexandre C. Pereira, Adriana M. Hung

**Affiliations:** 1 Division of Nephrology and Hypertension, Department of Medicine, Vanderbilt University Medical Center, Nashville, Tennessee; 2 Department of Biostatistics, Vanderbilt University Medical Center, Nashville, Tennessee; 3 Division of Quantitative Sciences, Department of Obstetrics and Gynecology, Vanderbilt University, Nashville, Tennessee; 4 Nashville VA Medical Center, VA Tennessee Valley Healthcare System, Nashville; 5 Million Veteran Program Coordinating Center, VA Boston Healthcare System, Boston, Massachusetts; 6 Department of Medicine, Brigham and Women’s Hospital and Harvard School of Medicine, Boston, Massachusetts; 7 VA Atlanta Health Care System, Decatur, Georgia; 8 Division of Endocrinology and Metabolism, Department of Medicine, Emory University School of Medicine, Atlanta, Georgia; 9 Vanderbilt Genetics Institute, Vanderbilt University Medical Center, Nashville, Tennessee; 10 Division of Epidemiology, Department of Medicine, Vanderbilt University Medical Center, Nashville, Tennessee

## Abstract

**Importance:**

Glucagon-like peptide 1 receptor agonists (GLP-1RAs) may have nephroprotective properties beyond those related to weight loss and glycemic control.

**Objective:**

To investigate the association of genetically proxied GLP-1RAs with kidney disease progression.

**Design, Setting, and Participants:**

This genetic association study assembled a national retrospective cohort of veterans aged 18 years or older from the US Department of Veterans Affairs Million Veteran Program between January 10, 2011, and December 31, 2021. Data were analyzed from November 2023 to February 2024.

**Exposures:**

Genetic risk score for systemic *GLP1R* gene expression that was calculated for each study participant based on genetic variants associated with *GLP1R* mRNA levels across all tissue samples within the Genotype-Tissue Expression project.

**Main Outcomes and Measures:**

The primary composite outcome was incident end-stage kidney disease or a 40% decline in estimated glomerular filtration rate. Cox proportional hazards regression survival analysis assessed the association between genetically proxied GLP-1RAs and kidney disease progression.

**Results:**

Among 353 153 individuals (92.5% men), median age was 66 years (IQR, 58.0-72.0 years) and median follow-up was 5.1 years (IQR, 3.1-7.2 years). Overall, 25.7% had diabetes, and 45.0% had obesity. A total of 4.6% experienced kidney disease progression. Overall, higher genetic *GLP1R* gene expression was associated with a lower risk of kidney disease progression in the unadjusted model (hazard ratio [HR], 0.96; 95% CI, 0.92-0.99; *P* = .02) and in the fully adjusted model accounting for baseline patient characteristics, body mass index, and the presence or absence of diabetes (HR, 0.96; 95% CI, 0.92-1.00; *P* = .04). The results were similar in sensitivity analyses stratified by diabetes or obesity status.

**Conclusions and Relevance:**

In this genetic association study, higher *GLP1R* gene expression was associated with a small reduction in risk of kidney disease progression. These findings support pleiotropic nephroprotective mechanisms of GLP-1RAs independent of their effects on body weight and glycemic control.

## Key Points

**Question:**

Are glucagon-like peptide 1 receptor agonists (GLP-1RAs) associated with kidney disease progression?

**Findings:**

In this genetic association study of 353 153 adults, higher genetic *GLP1R* gene expression as a proxy for GLP-1RAs was associated with a small reduction in the risk of kidney disease progression, even after adjusting for obesity and diabetes.

**Meaning:**

These findings support a nephroprotective role of GLP-1RAs.

## Teaser

This genetic association study investigates whether *GLP1R* gene expression as a proxy for glucagon-like peptide 1 receptor agonists is associated with reductions in kidney disease progression.

## Introduction

Glucagon-like peptide 1 receptor agonists (GLP-1RAs) are incretin mimetics indicated for the treatment of diabetes and obesity.^1,2^ GLP-1RAs may have nephroprotective properties beyond those related to glycemic control and weight loss.^3,4^ The FLOW trial, studying effects of semaglutide in patients with chronic kidney disease and type 2 diabetes, demonstrated the efficacy of a GLP-1RA in reducing kidney disease progression.^5^ However, the FLOW trial and similar studies have been limited to individuals with diabetes, leaving the efficacy of GLP-1RAs for reducing kidney disease progression in broader populations uncertain.^6,7^ The effect of genetic variants that influence GLP-1 receptor gene (*GLP1R*) expression may serve as a proxy for GLP-1RA treatment, providing a detailed assessment of its effects on kidney function in different clinical contexts.

Expression quantitative trait loci (eQTLs) are genetic variants that capture predicted levels of expression of a particular gene transcript in 1 or more tissues. Because eQTLs reflect germline genetic variation, they are less susceptible to biases typically found in observational studies, thus providing a better understanding of putatively causal molecular mechanisms underlying clinical outcomes.^8^ A previous study reported that *GLP1R* cell expression was associated with improved glucose control and adiposity, essentially mirroring the GLP-1RA drug effect.^9^ By comparing kidney outcomes in individuals with genetically determined higher vs lower *GLP1R* expression, genetic analyses can help evaluate the therapeutic potential of GLP-1RAs.

Our study assessed the association between genetically determined *GLP1R* gene expression in human tissues and kidney disease progression, leveraging the extensive biobank and genetic resources of the US Department of Veterans Affairs (VA) Million Veteran Program (MVP). Through a survival analysis, we examined the association of this genetic proxy for GLP-1RAs with kidney disease progression, performing sequential adjustments and subgroup analyses to estimate the influence of its metabolic effects. Our results may help the design and interpretation of contemporary GLP-1RA clinical trials.

## Methods

### Ethics and Protocols

All documents and protocols for this genetic association study were approved by the VA Central Institutional Review Board, the Tennessee Valley Healthcare System Office of Research & Development, and the MVP Publication and Presentation Committee. All MVP participants provided written consent. This study followed the Strengthening the Reporting of Genetic Association Studies (STREGA) reporting guideline.

### Study Population

The MVP is a research initiative by the VA with the objective to study the interaction between genetics, lifestyle behaviors, environmental factors, and health outcomes in over 1 million veterans.^10^ The MVP includes fully consented participants recruited from more than 63 VA medical facilities. Recruitment began in 2011, with participants completing baseline and lifestyle questionnaires, providing a blood sample, and granting access to their electronic health record.^11,12^ Blood samples were collected by phlebotomists and banked at the VA Central Biorepository in Boston, Massachusetts. Genotyping was performed using a customized Affymetrix Axiom biobank array (Thermo Fisher Scientific), with stringent quality control measures as previously described.^13^

**Figure 1.**
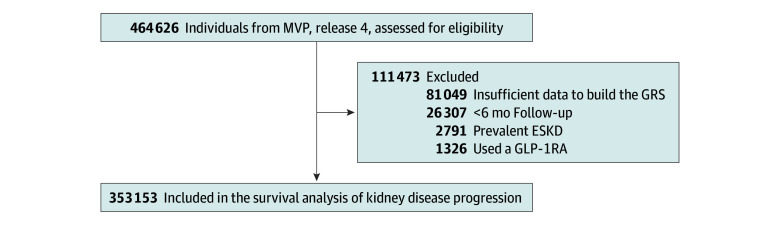
Flowchart Demonstrating the Selection of Study Participants for the Survival Analysis The national cohort was derived from the European Ancestry Veterans from the Million Veteran Program (MVP), release 4. ESKD indicates end-stage kidney disease; GLP-1RA, glucagon-like peptide 1 receptor agonist; GRS, genetic risk score.

This study included a retrospective cohort of veterans aged 18 years or older. Cohort entry was between January 10, 2011, and December 31, 2021, and the enrollment date was the start of follow-up. We excluded patients who had less than 6 months of follow-up, were receiving dialysis or had a kidney transplant prior to enrollment, or had received a prescription for a GLP-1RA within 365 days of enrollment (Figure 1).

### Exposure

#### Rationale

The exposure was a genetic risk score (GRS) for systemic *GLP1R* expression that was calculated for each study participant based on genetic variants associated with *GLP1R* mRNA levels within the Genotype-Tissue Expression Project (GTEx).^14^ In brief, GLP-1 is secreted by enteroendocrine L cells in the lower gastrointestinal tract in response to nutrient intake, acting in the pancreas to stimulate glucose-dependent insulin secretion and at other sites to reduce gastric emptying and food consumption.^15^ A prior study demonstrated that rare genetic variants linked to alterations in *GLP1R* cellular expression significantly affected adiposity and glycemic control, mirroring the anticipated effects of GLP-1RA treatments.^9^ In this study, we assessed common genetic variants associated with *GLP1R* gene expression to serve as a drug proxy for GLP-1RAs. Although these common genetic variants exhibit small effect sizes, especially when compared with the pronounced effects of pharmacologic GLP-1RA treatments, their association with clinical outcomes becomes evident when analyzed on a population scale.^16^

#### Genetic Instruments for *GLP1R* Expression

Genetic instruments for *GLP1R* expression were derived from GTEx, version 8, a comprehensive resource that reports eQTLs across human tissue types.^14^ The GTEx, version 8, release includes 15 201 RNA-sequencing samples from 49 tissue types from 838 individuals.^17,18^ Open access data were accessed via the GTEx Google Cloud Platform. We selected cis–gene-region genetic variants within 1 Mb of the transcription start of *GLP1R*. Recognizing the varied biological functions of GLP-1RA across different tissue types, we generated systemic genetic instruments by calculating the overall effect estimate for each cis–gene-region genetic variant across all available tissue samples in GTEx using a random-effects meta-analysis (eFigure 1 in Supplement 1).^19,20^ Estimates were weighted by both normalized transcript per million (TPM) values and inverse variance to account for effect size and variability in gene expression levels within each tissue sample. Normalized TPM values were calculated by dividing the median TPM of each tissue sample by the sum of the median TPMs across all tissue samples, thus representing the proportional contribution of each tissue sample to the total systemic effect (ie, the sum across all tissues = 1) (eTable 1 in Supplement 1). Significant genetic variants were identified based on the Benjamini-Hochberg false-discovery rate–adjusted *P* value threshold of .05 for all cis–gene-region variants.^21^ Annotation was completed with ANNOVAR software based on the human genome build hg38. Linkage disequilibrium clumping was performed within a 10-kb window cluster using the 1000 Genomes Project European superpopulation reference panel.^22^ We set a linkage disequilibrium threshold at *r*^2^ < 0.2 and used the PLINK clumping method to select genetic variants based on the lowest *P* value within each cluster using the ieugwasr package in R, version 4.2.2 (R Project for Statistical Computing).^23,24,25^ The genetic instruments for *GLP1R* expression were composed of statistically significant and independent genetic variants associated with *GLP1R* mRNA levels across all tissue samples, reflecting the systemic nature of GLP-1RA action (eTable 2 and eFigures 2 and 3 in Supplement 1).

#### Calculation of a GRS

As a proxy for GLP-1RAs, the systemic genetic instrument for *GLP1R* expression was used to calculate a GRS using a standard approach.^26^ To distinguish between high and low *GLP1R* expression using the GRS, patients were categorized in the top tertile or the bottom tertile based on the distribution of scores across all participants in the VA MVP. This approach provided nonoverlapping comparator groups and simulated the binary nature of pharmacologic interventions in which the effect of a drug is either present or not. The GRS for an individual *i* was calculated by summing the products of the number of risk alleles X*_ij_* and their respective effect estimates β*_j_* across *n* genetic variants associated with systemic gene expression as follows:


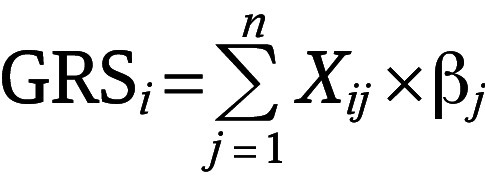
.

### Outcomes

The outcomes were assessed within the VA MVP, ensuring that the genetic instrument for systemic *GLP1R* expression and the outcomes dataset were derived from independent cohorts. The primary kidney composite outcome was defined as the incidence of ESKD or a 40% decline in eGFR, an established benchmark in nephrology clinical trials.^27^ The eGFR was calculated using each participant’s serum creatinine level using the race-free CKD Epidemiology Collaboration (CKD-EPI) equation.^28,29^ End-stage kidney disease was defined as an eGFR less than 15 mL/min/1.73 m^2^ on at least 2 occasions at least 3 months apart, by diagnosis codes for dialysis in the outpatient or inpatient setting on 2 occasions at least 3 months apart, or by diagnosis codes for kidney transplant (eTables 3 and 4 in Supplement 1).

### Covariates

Age at enrollment was computed from the date of birth. Sex was self-reported. We included individuals of European ancestry to be consistent with the GTEx dataset and adjusted analyses for the first 10 principal components (PCs) of ancestry using FlashPCA2.^30^ Determination of ancestry was based on a unified classification algorithm developed by the MVP. This algorithm integrates genetically inferred ancestry with self-identified race and ethnicity.^31^ This assignment has been extensively used in MVP publications and validated within several large biobanks.^13^ Baseline eGFR was calculated using the race-free, creatinine-based CKD-EPI equation from the laboratory visit at study enrollment.^29^ Blood pressure was taken from the closest outpatient clinic measurements within 365 days prior to enrollment. The use of an angiotensin-converting enzyme (ACE) inhibitor or angiotensin II receptor blocker (ARB) was determined from any outpatient prescription within 365 days prior to enrollment. Body mass index (BMI) was defined by dividing the weight in kilograms closest to time of study enrollment by the square of the height mode in meters based on all the measures of height available in the electronic health record. Diabetes was defined based on the outpatient prescription of any diabetes medication prior to enrollment plus 1 diagnostic code or based on the presence of at least 2 diagnosis codes for diabetes. Individuals who did not meet such criteria were considered to not have diabetes. All diagnosis code definitions are presented in eTable 4 in Supplement 1.

### Statistical Analysis

A Cox proportional hazards regression model was developed to assess the time in months until kidney disease progression using the top tertile vs the bottom tertile of the *GLP1R* GRS as the primary independent variable. Censoring occurred for participants who did not reach the end point of kidney failure for reasons including a participant’s last interaction with the health care system (as evidenced by the last vital signs recorded), end of the study period, or death. Sequential models were built to assess the association of *GLP1R* GRS with outcomes: (1) unadjusted (GRS), (2) minimally adjusted (GRS, age, sex, baseline eGFR, 10 PCs, blood pressure, and use of an ACE inhibitor or ARB), and (3) fully adjusted (minimally adjusted model plus the presence or absence of diabetes and BMI). Interaction terms between the *GLP1R* GRS and covariates were included to assess potential effect modification. The proportional hazards assumption was assessed through testing whether the scaled Schoenfeld residuals were correlated with time. A survival analysis was also performed in different subgroups defined by BMI and the presence or absence of diabetes. A significant association was considered at 2-sided *P* < .05. Survival analysis was performed using the survival and survminer packages in R, version 4.2.2.^32^ Data were analyzed from November 2023 to February 2024.

## Results

The VA MVP cohort included 353 153 individuals of European ancestry with a median age of 66.0 years (IQR, 58.0-72.0 years); 92.5% were men, and 7.5% were women. Overall, 25.7% of patients had diabetes, and 45.0% had obesity (Table). During a median follow-up period of 5.08 years (IQR, 3.15-7.16 years), 16 327 patients (4.6%) experienced kidney disease progression, defined as the composite outcome of ESKD or an eGFR decline of 40%.

**Table.** Baseline Characteristics of the VA Million Veteran Program Cohort

Characteristic	Patients^a^		
	All (N = 353 153)	No kidney disease progression (n = 336 826)^b^	Kidney disease progression (n = 16 327)^b^
Follow-up time, median (IQR), y^c^	5.08 (3.15-7.16)	5.16 (3.21-7.24)	3.61 (2.15-5.31)
Age, median (IQR), y	66.0 (58.0-72.0)	66.0 (57.0-72.0)	67.0 (63.0-73.0)
Sex			
Female	26 553 (7.5)	25 758 (7.7)	795 (4.9)
Male	326 599 (92.5)	311 067 (92.4)	15 532 (95.1)
Comorbidities			
Diabetes	90 728 (25.7)	81 304 (24.1)	9424 (57.7)
Obesity (BMI ≥30)	156 743 (45.0)	147 646 (44.5)	9097 (55.8)
BMI, median (IQR)	29.3 (26.0-33.4)	29.3 (26.0-33.3)	30.9 (27.1-35.3)
eGFR at baseline, median (IQR), mL/min/1.73 m^2^	79.4 (64.8-91.8)	79.8 (65.5-91.8)	71.2 (53.0-87.2)
HbA_1c_ level, median (IQR), %^d^	5.80 (5.50-6.50)	5.80 (5.50-6.40)	6.50 (5.80-7.70)
Blood pressure, median (IQR), mmHg^e^			
Systolic	129 (119-138)	129 (119-138)	133 (122-143)
Diastolic	76 (69-82)	76 (70-83)	74 (67-81)

Abbreviations: BMI, body mass index (calculated as weight in kilograms divided by height in meters squared); eGFR, estimated glomerular filtration rate; HbA_1c_, glycated hemoglobin; VA, US Department of Veterans Affairs.

^a^ Data are presented as number (percentage) of patients unless otherwise indicated.

^b^ Kidney disease progression was defined as an incident decline in eGFR of 40% or end-stage kidney disease.

^c^ Time to kidney disease progression, death, or the end of the study period.

^d^ Closest value measured within the prior 2 years.

^e^ Closest value measured within the past year.

**Figure 2.**
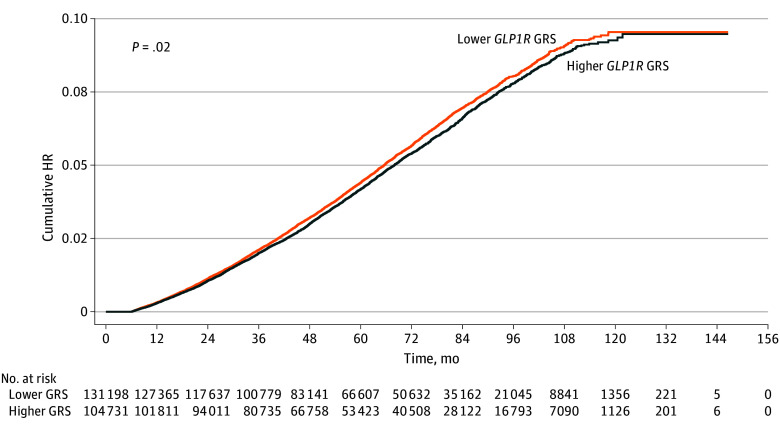
Cumulative Hazard Plot for Kidney Disease Progression, Stratified by High vs Low Genetic Risk Score (GRS) for *GLP1R* Expression Lower *GLP1R* expression represents the bottom tertile of GRS and higher expression, the top tertile. HR indicates hazard ratio.

**Figure 3.**
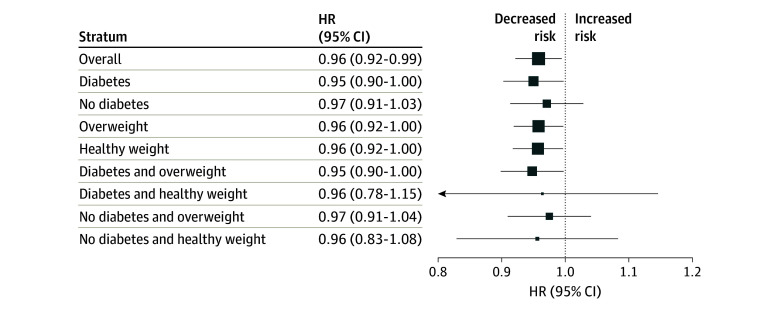
Subgroup Survival Analyses for the Association of Genetic *GLP1R* Expression With Risk of Kidney Disease Progression Analyses were adjusted for age, sex, 10 principal components of ancestry, baseline kidney function, use of an angiotensin-converting enzyme or angiotensin II receptor blocker, and blood pressure. The size of the data markers represents the sample size in each stratum.

In the survival analysis of *GLP1R* gene expression and kidney disease progression, higher genetic *GLP1R* expression determined by the GRS was associated with a lower risk of reaching the composite kidney outcome (Figure 2). This association was observed across multiple models, including the unadjusted model (hazard ratio [HR], 0.96; 95% CI, 0.92-0.99; *P* = .02); the minimally adjusted model accounting for age, sex, 10 PCs, baseline kidney function, blood pressure, and the use of an ACE inhibitor or ARB (HR, 0.96; 95% CI, 0.92-1.00; *P* = .03); and the fully adjusted model also accounting for BMI and the presence or absence of diabetes (HR, 0.96; 95% CI, 0.92-1.00; *P* = .04) (eTable 5 in Supplement 1). In subgroup analyses, the presence and direction of the association of genetic *GLP1R* expression with kidney disease progression was similar regardless of BMI or the presence or absence of diabetes (Figure 3 and eTable 6 in Supplement 1). When further stratifying by both diabetes and BMI, the HR for individuals with both diabetes and overweight or obesity was 0.95 (95% CI, 0.90-1.00; *P* = .04). In other subgroups, the results were not statistically significant but showed similar trends. Notably, there was no interaction effect between the *GLP1R* GRS and either diabetes (*P* = .98) or overweight or obesity (*P* = .47), indicating that the association of higher *GLP1R* expression with lower risk of kidney disease progression was consistent across subgroups.

## Discussion

GLP-1RAs improve glycemic control, reduce body weight, and protect against kidney disease progression.^7^ Our genetic findings support the hypothesis that higher *GLP1R* expression may have nephroprotective effects beyond outcomes related to weight loss and glycemic control. We studied naturally occurring genetic variation associated with systemic *GLP1R* expression and tested whether the same variants were also associated with a difference in kidney disease progression when examined on a population-wide scale. Critical to the interpretation of these findings is recognizing our focus on common genetic variants with small effect sizes, which reflected subtle but potentially meaningful associations with kidney disease progression.

Genetic proxies provide insight into drug mechanisms, with drugs supported by genetic evidence showing double the success rate in drug approval and repurposing pipelines.^33^ Clinical trials, such as the FLOW trial, demonstrated the efficacy of the GLP-1RA semaglutide in reducing kidney disease progression among patients with type 2 diabetes and chronic kidney disease with albuminuria, leading to an early halt of the trial due to a significant 24% reduction in major kidney disease events and decreased decline in eGFR among patients receiving semaglutide compared with placebo.^6,34,35^ The SELECT trial, although primarily a cardiovascular outcome study, suggested a potential kidney benefit in nondiabetic individuals with overweight or obesity.^36^ However, dedicated trials in populations without diabetes are still needed. Our study extends these findings by examining the nephroprotective potential of GLP-1RAs across a broad population. We demonstrated that higher genetic *GLP1R* expression was consistently associated with reduced kidney disease progression after adjusting for BMI and diabetes status. Subgroup analyses demonstrated similar presence and direction of associations in subgroups with healthy weight and without diabetes, indicating that these therapies could benefit a broader range of patients than currently recognized. Although larger sample sizes would be needed to detect statistically significant effects in these subgroups, our findings warrant further investigation and support the potential for broader clinical applications of GLP-1RAs.

### Strengths and Limitations

Our study has strengths. First, it leveraged individual-level data from the VA MVP, one of the largest biobanking initiatives worldwide, enabling us to study a longitudinal framework of kidney disease progression. The large sample size and extensive follow-up data allowed for the detection of modest effect sizes associated with common genetic variants, which would be obscured in smaller cohorts. Furthermore, we used a comprehensive analysis of eQTLs across multiple tissue types, simulating the systemic effects of *GLP1R* expression and reflecting the multitissue pharmacologic action of GLP-1RAs. In addition, our study used a clinically significant outcome—ESKD or a 40% decline in eGFR—that is a well-recognized benchmark in nephrology. The integration of clinical data, time-to-event analysis, and the significant clinical outcome increases the clinical relevance of our findings.

However, our study has limitations and assumptions. We constructed a GRS based on a linear additive model of genetic variants, which may oversimplify the complex genetic architecture underlying *GLP1R* expression. The translation of genetic associations to clinical applicability is a constant challenge. It is unknown whether small differences in risk based on genetic effects will be clinically significant. It is also unknown whether variation in receptor expression also corresponds to increased activation of the GLP-1 receptor. Additionally, our primary analysis was conducted among individuals of European ancestry due to the composition of the GTEx dataset, which may limit the generalizability of our findings to other populations. While the VA MVP cohort provides a large dataset with individual-level genetic data and longitudinal follow-up, replication of our findings in independent cohorts is needed. Nonetheless, the associations we have reported and their directionality offer insight into the mechanisms and therapeutic potential of GLP-1RAs.

## Conclusions

This genetic association study demonstrated that higher genetic *GLP1R* expression was associated with a small reduction in risk of kidney disease progression and supported a nephroprotective role of GLP-1RAs. Dedicated clinical trials to further validate the utility of GLP-1RAs in broader contexts are needed.

## Notes

Supplement 1. **eTable 1.** Adherence to Strengthening the Reporting of Genetic Association Studies (STREGA) Guidelines **eTable 2.** Weights of Normalized Transcript-per-Million Values for Meta-Analysis of Single Nucleotide Polymorphism Effect Estimates Across All Tissues **eTable 3.** Genetic Variants Associated With Systemic *GLP1R* Expression Used to Create the Genetic Risk Score **eTable 4.** Definition of End-Stage Kidney Disease **eTable 5.** Diagnosis Codes for Covariates **eTable 6.** Survival Analysis of the *GLP1R* Genetic Risk Score Effect on Kidney Disease Progression, Including Stepwise Adjustments for Covariates and Subgroup Analyses **eFigure 1.** Flowchart Detailing the Generation of a Systemic Genetic Instrument for *GLP1R* Expression From the Genotype-Tissue Expression Project **eFigure 2.** Distribution of Genetic Risk Scores for Systemic *GLP1R* Expression Among Study Participants **eFigure 3.** Linkage Disequilibrium Matrix for the Genetic Variants Associated With Systemic *GLP1R* Expression Used to Create the Genetic Risk Score **eAppendix.** VA Million Veteran Program—Core Acknowledgment for Publications (February 2024)

Supplement 2.
Nonauthor Collaborators

Supplement 3.
Data Sharing Statement
