# Supplementary material for: GLP1R Gene Expression and Kidney Disease Progression
Source: JAMA Netw Open. 2024 Oct 25;7(10):e2440286. doi: 10.1001/jamanetworkopen.2024.40286 (PMC11581634; doi:10.1001/jamanetworkopen.2024.40286)
Supplement: Supplement 1. — eTable 1. Adherence to Strengthening the Reporting of Genetic Association Studies (STREGA) Guidelines eTable 2. Weights of Normalized Transcript-per-Million Values for Meta-Analysis of Single Nucleotide Polymorphism Effect Estimates Across All Tissues eTable 3. Genetic Variants Associated With Systemic GLP1R Expression Used to Create the Genetic Risk Score eTable 4. Definition of End-Stage Kidney Disease eTable 5. Diagnosis Codes for Covariates eTable 6. Survival Analysis of the GLP1R Genetic Risk Score Effect on Kidney Disease Progression, Including Stepwise Adjustments for Covariates and Subgroup Analyses eFigure 1. Flowchart Detailing the Generation of a Systemic Genetic Instrument for GLP1R Expression From the Genotype-Tissue Expression Project eFigure 2. Distribution of Genetic Risk Scores for Systemic GLP1R Expression Among Study Participants eFigure 3. Linkage Disequilibrium Matrix for the Genetic Variants Associated With Systemic GLP1R Expression Used to Create the Genetic Risk Score eAppendix. VA Million Veteran Program—Core Acknowledgment for Publications (February 2024) [file jamanetwopen-e2440286-s001.pdf]

Supplementary Online Content

Triozzi JL, Yu Z, Giri A, et al; for the VA Million Veteran Program. *GLP1R* gene expression and kidney disease progression. *JAMA Netw Open*. 2024;7(10):e2440286. doi:10.1001/jamanetworkopen.2024.40286

eTable 1. Weights of Normalized Transcript per Million Values for Meta-Analysis of Single Nucleotide Polymorphism Effect Estimates Across All Tissues

eTable 2. Genetic Variants Associated With Systemic *GLP1R* Expression Used to Create the Genetic Risk Score

eTable 3. Definition of End-Stage Kidney Disease

eTable 4. Diagnosis Codes for Covariates

eTable 5. Survival Analysis of the *GLP1R* Genetic Risk Score Effect on Kidney Disease Progression, Including Stepwise Adjustments for Covariates and Subgroup Analyses

eFigure 1. Flowchart Detailing the Generation of a Systemic Genetic Instrument for *GLP1R* Expression From the Genotype-Tissue Expression Project

eFigure 2. Distribution of Genetic Risk Scores for Systemic *GLP1R* Expression Among Study Participants

eFigure 3. Linkage Disequilibrium Matrix for the Genetic Variants Associated With Systemic *GLP1R* Expression Used to Create the Genetic Risk Score

eAppendix. VA Million Veteran Program—Core Acknowledgement for Publications (February 2024)

This supplementary material has been provided by the authors to give readers additional information about their work.

© 2024 Triozzi JL et al. *JAMA Network Open*.

eTable 1. Weights of normalized transcript per million values for meta-analysis of single nucleotide polymorphism effect estimates across all tissues.

Tissue	TPM
Adipose Subcutaneous	0.005347
Adipose Visceral Omentum	0.004417
Adrenal Gland	0.00238
Artery Aorta	0
Artery Coronary	0.004562
Artery Tibial	0.010602
Brain Amygdala	0.001507
Brain Anterior cingulate cortex BA24	0.004436
Brain Caudate basal ganglia	0.038985
Brain Cerebellar Hemisphere	0.003724
Brain Cerebellum	0.00421
Brain Cortex	0.00981
Brain Frontal Cortex BA9	0.00401
Brain Hippocampus	0.003104
Brain Hypothalamus	0.029825
Brain Nucleus accumbens basal ganglia	0.021023
Brain Putamen basal ganglia	0.018393
Brain Spinal cord cervical c1	0.002074
Brain Substantia nigra	0
Breast Mammary Tissue	0.010591
Cells Cultured fibroblasts	0
Cells EBV transformed lymphocytes	0
Colon Sigmoid	0.001191
Colon Transverse	0.002594
Esophagus Gastroesophageal Junction	0.000986
Esophagus Mucosa	0.002147
Esophagus Muscularis	0
Heart Atrial Appendage	0.132596

© 2024 Triozzi JL et al. *JAMA Network Open*.

Heart Left Ventricle	0.044741
Kidney Cortex	0.003166
Liver	0
Lung	0.024889
Minor Salivary Gland	0.019252
Muscle Skeletal	0
Nerve Tibial	0.06929
Ovary	0
Pancreas	0.355041
Pituitary	0.002163
Prostate	0.007568
Skin Not Sun Exposed Suprapubic	0.01113
Skin Sun Exposed Lower leg	0.016588
Small Intestine Terminal Ileum	0.005497
Spleen	0.002499
Stomach	0.03184
Testis	0.058527
Thyroid	0.024682
Uterus	0.001873
Vagina	0.001368
Whole Blood	0.001372

Normalized TPM values were calculated by dividing the median TPM of each tissue by the sum of the median TPMs across all tissues, thus representing each tissue's proportional contribution to the total systemic effect (i.e., the sum across all tissues = 1.0).

Abbreviations: TPM—transcript per million.

© 2024 Triozzi JL et al. *JAMA Network Open*.

eTable 2. Genetic variants associated with systemic *GLP1R* expression used to create the genetic risk score.

Chr	Pos	NEA	EA	TSS	Beta	SE	*p*-value
6	39048860	C	T	62	0.152523	0.036477	2.90E-05
6	39073305	A	G	24507	-0.29631	0.090058	0.001001
6	39011665	G	C	-37133	-0.18453	0.040686	5.75E-06
6	39264926	G	A	216128	0.284428	0.090481	0.001669
6	39128276	G	C	79478	-0.25225	0.077073	0.001065
6	39051177	G	A	2379	-0.28656	0.065503	1.22E-05
6	39808404	A	G	759606	-0.08121	0.022217	0.000257
6	38237522	C	T	-811276	0.226757	0.073015	0.001899
6	38363354	T	A	-685444	-0.3703	0.094072	8.27E-05
6	39615359	T	C	566561	0.1659	0.049471	0.000798
6	39327729	G	T	278931	-0.12515	0.041186	0.002376
6	38927373	G	C	-121425	0.083492	0.026902	0.001912
6	39381589	G	A	332791	0.119934	0.03203	0.000181
6	39591175	A	C	542377	0.085053	0.022002	0.000111
6	39397525	T	C	348727	0.172934	0.055966	0.002002

Abbreviations: Chr—chromosome; Pos—position; NEA—non-effect allele; EA—effect allele; TSS—transcription start site distance; Beta—effect size; SE—standard error.

© 2024 Triozzi JL et al. *JAMA Network Open*.

eTable 3. Definition of end-stage kidney disease.

Outcome	Definition
eGFR < 15 ml/min/1.73m2	An outpatient laboratory measurement of eGFR < 15 mL/min/1.73 m^2 with confirmatory eGFR < 15 at least 3 months apart.
Dialysis	Either 2 outpatient codes for dialysis or 1 outpatient and 1 inpatient diagnosis code dialysis in the primary position at least 3 months apart.
Transplant	Either an outpatient code for renal transplant or an inpatient code for renal transplant positioned as the primary diagnosis. In the outpatient setting two codes are required on two different dates.

Abbreviations: eGFR—estimated glomerular filtration rate.

© 2024 Triozzi JL et al. *JAMA Network Open*.

eTable 4. Diagnosis codes for covariates.

**Dialysis**	
ICD-9	V45.1, V45.11, V56.0, V56.1, V56.2, V56.8, 585.6, V45.12, V56.31, V56.32, 792.5, 39.95, 39.952, 54.982, 39.953, 54.98, 54.983, 99.78
ICD-10	N18.6 R88.0 Z49.31, Z49.32, Z91.15,3E1M39Z Z49.01, Z49.02, Z99.2
ICD9P C	39.951, 54.981
CPT	90941, 90942, 90943, 90944, G0491, G0492, M0916, M0920, M0931, M0932, 90996, A4690, A4750, A4755, 90935, 90937, 90947, 90999, 90988, 90997, 4054F, 4055F, E1590, G8715, G8727, M0936, 36516, 36901, 36902, 36903, 36904, 36905, 36906, 36516, 36901, 36902, 36903, 36904, 36905, 36906, 36907, 36908, 36909, 4052F, 4053F, 90939, 90940, 90963, 90964, 90965 ,90966, 90967, 90968, 90969, 90970, 90976, 90977, 90978, 90979, 90982, 90983, 90984, 90985, 90991, 90994, 99512, 99559, G0257, G0320, G0321, G0322, G0323, G0327, G8075, G8076, G8714, G8956, G9264, G9265, G9266, M0923, M0928, M0937, M0940, M0944, M0945, M0948, M0952, M0956, S9335, S9339, G9240, G9241, 0505F, 0507F, 9092, 90921, 90924, 90925,90951, 90952, 90953, 90954, 90955, 90956, 90957, 90958, 90959, 90960, 90961, 90962, 90989, 90990, 90992, 90993, 90995, 90998, 4054F, 4055F
**Diabetes**	
ICD-9	250.1, 250.12, 250.21, 250.4, 250.41, 250.43, 250.5, 250.51, 250.6, 250.63, 250.72, 250.83, 250.93, 357.2, 362.03, 250, 250.22, 250.23, 250.3, 250.31, 250.53, 250.91, 362.01, 362.04, 362.06, 362.07, V53.91, 250.01, 250.03, 250.13, 250.2, 250.42, 250.52, 250.73, 250.82, 250.9, 250.92, 362.02, 362.05, 366.41, 250.11, 250.32, 250.33, 250.61, 250.62, 250.7, 250.71, 250.8, 250.81, V45.85 250.02
ICD-10	E11.311, E10.42, E11.319, E11.628, E11.69, E10.618, E10.9, E10.37X3, E10.37X2, E10.11, E10.638, E11.3493, E11.8, E10.319, E11.3592, E11.3391, E10.69, E11.10, E10.29, E10.37X9, E11.9, E10.621, E11.3492, E09.36, E11.3399, E11.11, E11.3591, E11.3299, E10.620, E11.638, E09.42, E10.10, E10.65, E11.3499, E11.01, E11.630, E10.630, E08.36, E10.51, E11.649, E11.621, E11.620, E11.39, E10.21, E10.8, E11.65, E11.51, E10.40, Z46.81, E11.3393, E10.39, E13.42, E11.3593, E10.641, E10.311, E11.36, E11.3292, E10.649, E11.3491, E11.622, E11.3291, E11.618, E11.3599, E10.36, E11.00, E08.42, E13.36, Z96.41, E11.29, E11.3293, E10.628, E11.40, E13.10, E10.622, E11.641, E10.37X1, E11.42, E11.3392, E11.21

Abbreviations: ICD—International Classification of Diseases; CPT—Current Procedural Terminology.

© 2024 Triozzi JL et al. *JAMA Network Open*.

eTable 5. Survival analysis of the *GLP1R* genetic risk score effect on kidney disease progression, including stepwise adjustments for covariates and subgroup analyses.

**Overall**				
	N	HR	95% CI	*p*-value
Model 1	353153	0.956	0.921 – 0.993	**0.022**
Model 2	315815	0.958	0.922 – 0.995	**0.028**
Model 3	315509	0.961	0.925 – 0.999	**0.042**
**Diabetes**				
	N	HR	95% CI	*p*-value
Model 1	90728	0.951	0.905 – 1.000	0.051
Model 2	86216	0.949	0.902 – 0.998	**0.041**
**No diabetes**				
	N	HR	95% CI	*p*-value
Model 1	262425	0.969	0.914 – 1.028	0.295
Model 2	229599	0.975	0.919 – 1.035	0.404
**Overweight/obese**				
	N	HR	95% CI	*p*-value
Model 1	284713	0.952	0.914 – 0.992	**0.019**
Model 2	258729	0.957	0.918 – 0.997	**0.037**
**Normal weight**				
	N	HR	95% CI	*p*-value
Model 1	63482	0.985	0.887 – 1.094	0.777
Model 2	56780	0.969	0.871 – 1.078	0.560
**Diabetes & overweight/obese**				
	N	HR	95% CI	*p*-value
Model 1	82831	0.949	0.901 – 0.999	**0.047**
Model 2	78914	0.947	0.899 – 0.998	**0.044**
**Diabetes & normal weight**				
	N	HR	95% CI	*p*-value
Model 1	7709	0.988	0.822 – 1.186	0.893
Model 2	7267	0.973	0.808 – 1.172	0.771

© 2024 Triozzi JL et al. *JAMA Network Open*.

No diabetes & overweight/obese				
	N	HR	95% CI	*p*-value
Model 1	201882	0.963	0.901 – 1.028	0.258
Model 2	179815	0.974	0.911 – 1.041	0.440
**No diabetes & normal weight**				
	N	HR	95% CI	*p*-value
Model 1	55773	0.987	0.869 – 1.122	0.845
Model 2	49513	0.975	0.856 – 1.110	0.698

The hazard ratio for kidney disease progression represents the top tertile versus the bottom tertile of the genetic risk score for systemic *GLP1R* expression. Model 1 is unadjusted. Model 2 is adjusted for age, sex, the first 10 principal components, systolic blood pressure, and the use of an ACE or ARB. Model 3 is Model 2 plus adjustments for body mass index and the presence or absence of diabetes.

© 2024 Triozzi JL et al. *JAMA Network Open*.

eFigure 1. Flowchart detailing the generation of a systemic genetic instrument for GLP1R expression from the Genotype-Tissue Expression Project.

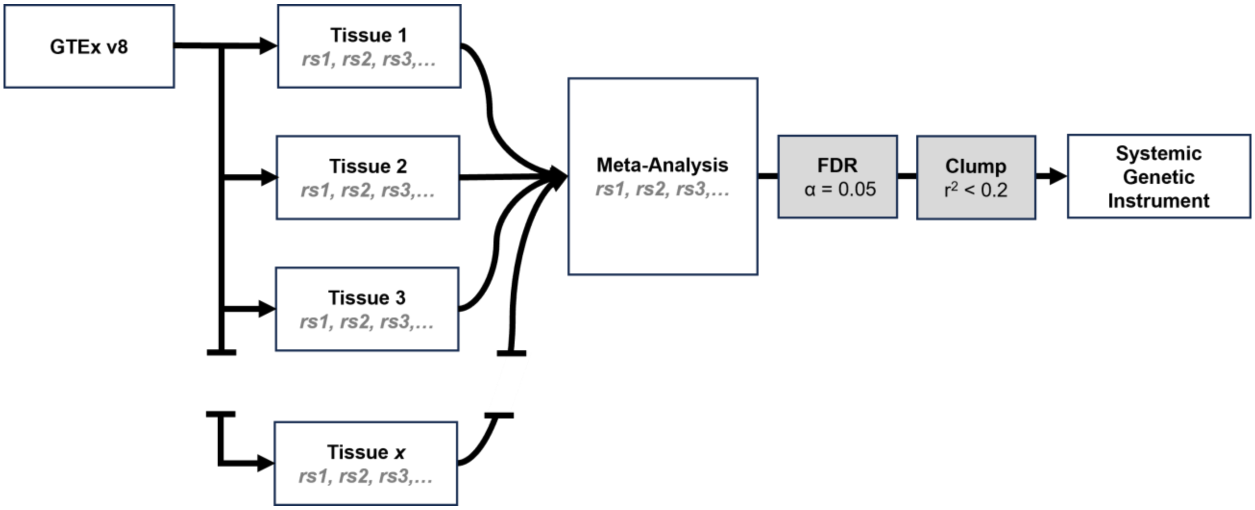

Abbreviations: GTEx v8—The Genotype-Tissue Expression project version 8; FDR—false discovery rate.

© 2024 Triozzi JL et al. *JAMA Network Open*.

eFigure 2. Distribution of genetic risk scores for systemic *GLP1R* expression among study participants.

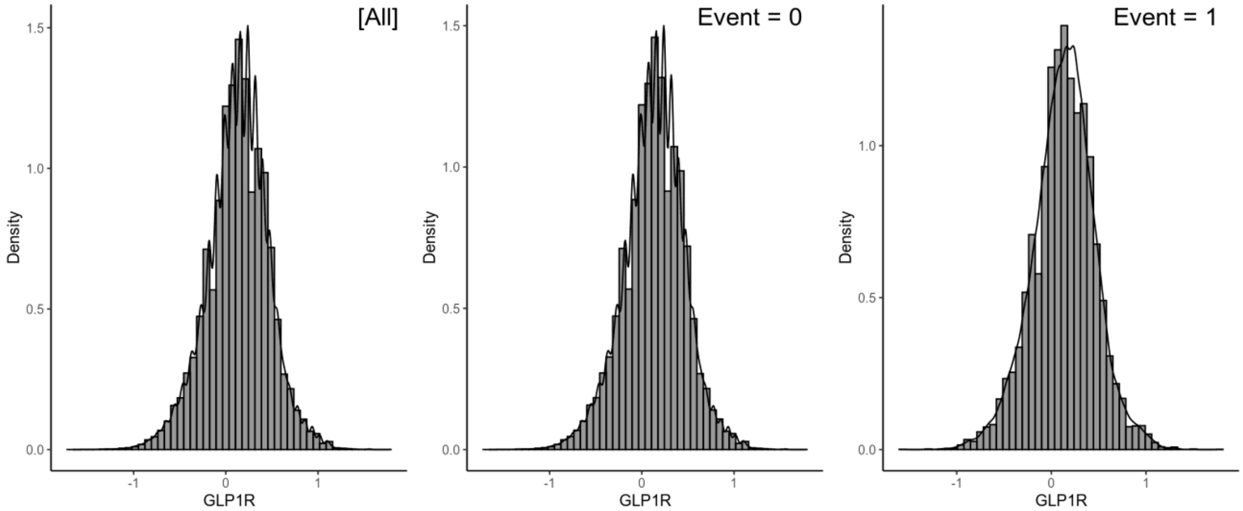

Event corresponds to kidney disease progression, defined as the incident decline in eGFR of 40% or end-stage kidney disease during the follow-up period.

© 2024 Triozzi JL et al. *JAMA Network Open*.

eFigure 3. Linkage disequilibrium matrix for the genetic variants associated with systemic *GLP1R* expression used to create the genetic risk score.

RSID	rs16890468	rs180699747	rs6933584	rs10484850	rs10305420	rs13219169	rs10305476	rs116018552	rs115530377	rs60567936	rs716952	rs80022279	rs7744341	rs2045577	rs13220608
rs16890468	1	0	0.004	0	0	0.001	0	0.001	0.001	0.001	0	0	0.003	0	0.004
rs180699747	0	1	0	0.001	0.002	0.001	0	0	0	0	0	0.001	0.004	0.001	0.001
rs6933584	0.004	0	1	0.067	0.044	0.018	0.005	0	0	0.01	0.001	0.002	0	0	0.006
rs10484850	0	0.001	0.067	1	0.16	0.082	0.013	0.006	0.001	0.002	0.003	0.001	0	0	0
rs10305420	0	0.002	0.044	0.16	1	0.03	0.007	0	0	0	0.001	0	0.005	0.005	0.003
rs13219169	0.001	0.001	0.018	0.082	0.03	1	0	0.082	0.001	0.014	0	0.001	0.001	0	0
rs10305476	0	0	0.005	0.013	0.007	0	1	0	0.001	0	0	0.001	0.009	0.003	0
rs116018552	0.001	0	0	0.006	0	0.082	0	1	0.001	0.002	0.001	0.001	0.001	0.003	0.002
rs115530377	0.001	0	0	0.001	0	0.001	0.001	0.001	1	0	0.002	0	0.005	0	0
rs60567936	0.001	0	0.01	0.002	0	0.014	0	0.002	0	1	0.001	0	0	0	0.003
rs716952	0	0	0.001	0.003	0.001	0	0	0.001	0.002	0.001	1	0.133	0	0	0.002
rs80022279	0	0.001	0.002	0.001	0	0.001	0.001	0.001	0	0	0.133	1	0.013	0.076	0.005
rs7744341	0.003	0.004	0	0	0.005	0.001	0.009	0.001	0.005	0	0	0.013	1	0.173	0.046
rs2045577	0	0.001	0	0	0.005	0	0.003	0.003	0	0	0	0.076	0.173	1	0.01
rs13220608	0.004	0.001	0.006	0	0.003	0	0	0.002	0	0.003	0.002	0.005	0.046	0.01	1

The plot illustrates pairwise linkage disequilibrium between the genetic variants associated with systemic GLP1R expression based on the European reference population. These were generated using the National Institutes of Health LDlink. The colors correspond to R^2 values, where white indicates no correlation and red indicates absolute correlation.

© 2024 Triozzi JL et al. *JAMA Network Open*.

eAppendix. VA Million Veteran Program: Core Acknowledgement for Publications (February 2024).

MVP Program Office
Sumitra Muralidhar, Ph.D., Program Director
US Department of Veterans Affairs, 810 Vermont Avenue NW, Washington, DC 20420
Jennifer Moser, Ph.D., Associate Director, Scientific Programs
US Department of Veterans Affairs, 810 Vermont Avenue NW, Washington, DC 20420
Jennifer E. Deen, B.S., Associate Director, Cohort & Public Relations
US Department of Veterans Affairs, 810 Vermont Avenue NW, Washington, DC 20420

MVP Executive Committee
Co-Chair: Philip S. Tsao, Ph.D.
VA Palo Alto Health Care System, 3801 Miranda Avenue, Palo Alto, CA 94304
Co-Chair: Sumitra Muralidhar, Ph.D.
US Department of Veterans Affairs, 810 Vermont Avenue NW, Washington, DC 20420
J. Michael Gaziano, M.D., M.P.H.
VA Boston Healthcare System, 150 S. Huntington Avenue, Boston, MA 02130
Elizabeth Hauser, Ph.D.
Durham VA Medical Center, 508 Fulton Street, Durham, NC 27705
Amy Kilbourne, Ph.D., M.P.H.
VA HSR&D, 2215 Fuller Road, Ann Arbor, MI 48105
Michael Matheny, M.D., M.S., M.P.H.
VA Tennessee Valley Healthcare System, 1310 24th Ave. South, Nashville, TN 37212
Dave Oslin, M.D.
Philadelphia VA Medical Center, 3900 Woodland Avenue, Philadelphia, PA 19104

MVP Co-Principal Investigators
J. Michael Gaziano, M.D., M.P.H.
VA Boston Healthcare System, 150 S. Huntington Avenue, Boston, MA 02130
Philip S. Tsao, Ph.D.
VA Palo Alto Health Care System, 3801 Miranda Avenue, Palo Alto, CA 94304

MVP Core Operations
Jessica V. Brewer, M.P.H., Director, MVP Recruitment & Enrollment
VA Boston Healthcare System, 150 S. Huntington Avenue, Boston, MA 02130
Mary T. Brophy M.D., M.P.H., Director, VA Central Biorepository
VA Boston Healthcare System, 150 S. Huntington Avenue, Boston, MA 02130
Kelly Cho, M.P.H, Ph.D., Director, MVP Phenomics Data Core
VA Boston Healthcare System, 150 S. Huntington Avenue, Boston, MA 02130
Lori Churby, B.S., Director, MVP Regulatory Affairs
VA Palo Alto Health Care System, 3801 Miranda Avenue, Palo Alto, CA 94304
Scott L. DuVall, Ph.D., Director, VA Informatics and Computing Infrastructure (VINCI)
VA Salt Lake City Health Care System, 500 Foothill Drive, Salt Lake City, UT 84148
Saiju Pyarajan Ph.D., Director, Data and Computational Sciences
VA Boston Healthcare System, 150 S. Huntington Avenue, Boston, MA 02130

© 2024 Triozzi JL et al. *JAMA Network Open*.

Luis E. Selva, Ph.D., Executive Director, MVP Biorepositories
VA Boston Healthcare System, 150 S. Huntington Avenue, Boston, MA 02130
Shahpoor (Alex) Shayan, M.S., Director, MVP Recruitment and Enrollment Informatics
VA Boston Healthcare System, 150 S. Huntington Avenue, Boston, MA 02130
Stacey B. Whitbourne, Ph.D., Director, MVP Cohort Management
VA Boston Healthcare System, 150 S. Huntington Avenue, Boston, MA 02130
MVP Coordinating Centers
MVP Coordinating Center, Boston - J. Michael Gaziano, M.D., M.P.H.
VA Boston Healthcare System, 150 S. Huntington Avenue, Boston, MA 02130
MVP Coordinating Center, Palo Alto – Philip S. Tsao, Ph.D.
VA Palo Alto Health Care System, 3801 Miranda Avenue, Palo Alto, CA 94304
MVP Information Center, Canandaigua – Brady Stephens, M.S.
Canandaigua VA Medical Center, 400 Fort Hill Avenue, Canandaigua, NY 14424
Cooperative Studies Program Clinical Research Pharmacy Coordinating Center, Albuquerque – Todd Connor, Pharm.D.; Dean P. Argyres, B.S., M.S.
New Mexico VA Health Care System, 1501 San Pedro Drive SE, Albuquerque, NM 87108

MVP Publications and Presentations Committee
Co-Chair: Themistocles L. Assimes, M.D., Ph. D
VA Palo Alto Health Care System, 3801 Miranda Avenue, Palo Alto, CA 94304
Co-Chair: Adriana Hung, M.D.; M.P.H
VA Tennessee Valley Healthcare System, 1310 24th Ave. South, Nashville, TN 37212
Co-Chair: Henry Kranzler, M.D.
Philadelphia VA Medical Center, 3900 Woodland Avenue, Philadelphia, PA 19104

MVP Local Site Investigators
Samuel Aguayo, M.D., Phoenix VA Health Care System
650 E. Indian School Road, Phoenix, AZ 85012
Sunil Ahuja, M.D., South Texas Veterans Health Care System
7400 Merton Minter Boulevard, San Antonio, TX 78229
Kathrina Alexander, M.D., Veterans Health Care System of the Ozarks
1100 North College Avenue, Fayetteville, AR 72703
Xiao M. Androulakis, M.D., Columbia VA Health Care System
6439 Garners Ferry Road, Columbia, SC 29209
Prakash Balasubramanian, M.D., William S. Middleton Memorial Veterans Hospital
2500 Overlook Terrace, Madison, WI 53705
Zuhair Ballas, M.D., Iowa City VA Health Care System
601 Highway 6 West, Iowa City, IA 52246-2208
Elizabeth S. Bast, M.D., M.P.H., Miami VA Health Care System
1201 NW 16th Street, 11 GRC, Miami FL 33125
Jean Beckham, Ph.D., Durham VA Medical Center
508 Fulton Street, Durham, NC 27705
Sujata Bhushan, M.D., VA North Texas Health Care System
4500 S. Lancaster Road, Dallas, TX 75216
Edward Boyko, M.D., VA Puget Sound Health Care System

© 2024 Triozzi JL et al. *JAMA Network Open*.

1660 S. Columbian Way, Seattle, WA 98108-1597
David Cohen, M.D., Portland VA Medical Center
3710 SW U.S. Veterans Hospital Road, Portland, OR 97239
Louis Dellitalia, M.D., Birmingham VA Medical Center
700 S. 19th Street, Birmingham AL 35233
Gerald Wayne Dryden, Jr., M.D., Ph.D., Louisville VA Medical Center
800 Zorn Avenue, Louisville, KY 40206
L. Christine Faulk, M.D., Robert J. Dole VA Medical Center
5500 East Kellogg Drive, Wichita, KS 67218-1607
Joseph Fayad, M.D., VA Southern Nevada Healthcare System
6900 North Pecos Road, North Las Vegas, NV 89086
Daryl Fujii, Ph.D., VA Pacific Islands Health Care System
459 Patterson Rd, Honolulu, HI 96819
Saib Gappy, M.D., John D. Dingell VA Medical Center
4646 John R Street, Detroit, MI 48201
Frank Gesek, Ph.D., White River Junction VA Medical Center
163 Veterans Drive, White River Junction, VT 05009
Michael Godschalk, M.D., Richmond VA Medical Center
1201 Broad Rock Blvd., Richmond, VA 23249
Jennifer Greco, M.D., Sioux Falls VA Health Care System
2501 W 22nd Street, Sioux Falls, SD 57105
Todd W. Gress, M.D., Ph.D., Hershel “Woody” Williams VA Medical Center
1540 Spring Valley Drive, Huntington, WV 25704
Samir Gupta, M.D., M.S.C.S., VA San Diego Healthcare System
3350 La Jolla Village Drive, San Diego, CA 92161
Salvador Gutierrez, M.D., Edward Hines, Jr. VA Medical Center
5000 South 5th Avenue, Hines, IL 60141
Mark Hamner, M.D., Ralph H. Johnson VA Medical Center
109 Bee Street, Mental Health Research, Charleston, SC 29401
John Harley, M.D., Ph.D., Cincinnati VA Medical Center
3200 Vine Street, Cincinnati, OH 45220
Daniel J. Hogan, M.D., Bay Pines VA Healthcare System
10,000 Bay Pines Blvd Bay Pines, FL 33744
Adriana Hung, M.D., M.P.H., VA Tennessee Valley Healthcare System
1310 24th Avenue, South Nashville, TN 37212
Robin Hurley, M.D., W.G. (Bill) Hefner VA Medical Center
1601 Brenner Ave, Salisbury, NC 28144
Pran Iruvanti, D.O., Ph.D., Hampton VA Medical Center
100 Emancipation Drive, Hampton, VA 23667
Frank Jacono, M.D., VA Northeast Ohio Healthcare System
10701 East Boulevard, Cleveland, OH 44106
Darshana Jhala, M.D., Philadelphia VA Medical Center
3900 Woodland Avenue, Philadelphia, PA 19104
Seema Joshi, M.D., F.A.C.P., ABOIM; VA Eastern Kansas Health Care System
4101 S 4th Street Trafficway, Leavenworth, KS 66048
Scott Kinlay, M.B.B.S., Ph.D., VA Boston Healthcare System

© 2024 Triozzi JL et al. *JAMA Network Open*.

150 S. Huntington Avenue, Boston, MA 02130
Michael Landry, Ph.D., Southeast Louisiana Veterans Health Care System
2400 Canal Street, New Orleans, LA 70119
Peter Liang, M.D., M.P.H., VA New York Harbor Healthcare System
423 East 23rd Street, New York, NY 10010
Suthat Liangpunsakul, M.D., M.P.H., Richard Roudebush VA Medical Center
1481 West 10th Street, Indianapolis, IN 46202
Jack Lichy, M.D., Ph.D., Washington DC VA Medical Center
50 Irving St, Washington, D. C. 20422
Tze Shien Lo, M.D., Fargo VA Health Care System
2101 N. Elm, Fargo, ND 58102
C. Scott Mahan, M.D., Charles George VA Medical Center
1100 Tunnel Road, Asheville, NC 28805
Ronnie Marrache, M.D., VA Maine Healthcare System Center, Augusta, ME 04330
Stephen Mastorides, M.D., James A. Haley Veterans' Hospital
13000 Bruce B. Downs Blvd, Tampa, FL 33612
Kristin Mattocks, Ph.D., M.P.H., Central Western Massachusetts Healthcare System
421 North Main Street, Leeds, MA 01053
Paul Meyer, M.D., Ph.D., Southern Arizona VA Health Care System
3601 S 6th Avenue, Tucson, AZ 85723
Jonathan Moorman, M.D., Ph.D., James H. Quillen VA Medical Center
Corner of Lamont & Veterans Way, Mountain Home, TN 37684
Providencia Morales, R.N., Northern Arizona VA Health Care System
500 Highway 89 North, Prescott, AZ 86313
Timothy Morgan, M.D., VA Long Beach Healthcare System
5901 East 7th Street Long Beach, CA 90822
Maureen Murdoch, M.D., M.P.H., Minneapolis VA Health Care System
One Veterans Drive, Minneapolis, MN 55417
Eknath Naik, M.D., Ph.D., West Palm Beach VA Medical Center,
7305 North Military Trail, West Palm Beach, FL 33410-6400
James Norton, Ph.D., VA Health Care Upstate New York
113 Holland Avenue, Albany, NY 12208
Olaoluwa Okusaga, M.D., Michael E. DeBakey VA Medical Center
2002 Holcombe Blvd, Houston, TX 77030
Michael K. Ong, M.D., VA Greater Los Angeles Health Care System
11301 Wilshire Blvd, Los Angeles, CA 90073
Kris Ann Oursler, M.D., Salem VA Medical Center
1970 Roanoke Blvd, Salem, VA 24153
Ismene Petrakis, M.D., VA Connecticut Healthcare System
950 Campbell Avenue, West Haven, CT 06516
Samuel Poon, M.D., Manchester VA Medical Center
718 Smyth Road, Manchester, NH 03104
Amneet S. Rai, Pharm.D., VA Sierra Nevada Health Care System
975 Kirman Avenue, Reno, NV 89502
Michael Rauchman, M.D., St. Louis VA Health Care System
915 North Grand Blvd, St. Louis, MO 63106

© 2024 Triozzi JL et al. *JAMA Network Open*.

Richard Servatius, Ph.D., Syracuse VA Medical Center
800 Irving Avenue, Syracuse, NY 13210
Satish Sharma, M.D., Providence VA Medical Center
830 Chalkstone Avenue, Providence, RI 02908
River Smith, Ph.D., Eastern Oklahoma VA Health Care System
1011 Honor Heights Drive, Muskogee, OK 74401
Peruvemba Sriram, M.D., N. FL/S. GA Veterans Health System
1601 SW Archer Road, Gainesville, FL 32608
Patrick Strollo, Jr., M.D., VA Pittsburgh Health Care System
University Drive, Pittsburgh, PA 15240
Neeraj Tandon, M.D., Overton Brooks VA Medical Center
510 East Stoner Ave, Shreveport, LA 71101
Philip Tsao, Ph.D., VA Palo Alto Health Care System
3801 Miranda Avenue, Palo Alto, CA 94304-1290
Gerardo Villareal, M.D., New Mexico VA Health Care System
1501 San Pedro Drive, S.E. Albuquerque, NM 87108
Jessica Walsh, M.D., VA Salt Lake City Health Care System
500 Foothill Drive, Salt Lake City, UT 84148
John Wells, Ph.D., Edith Nourse Rogers Memorial Veterans Hospital
200 Springs Road, Bedford, MA 01730
Jeffrey Whittle, M.D., M.P.H., Clement J. Zablocki VA Medical Center
5000 West National Avenue, Milwaukee, WI 53295
Mary Whooley, M.D., San Francisco VA Health Care System
4150 Clement Street, San Francisco, CA 94121
Peter Wilson, M.D., Atlanta VA Medical Center
1670 Clairmont Road, Decatur, GA 30033
Junzhe Xu, M.D., VA Western New York Healthcare System
3495 Bailey Avenue, Buffalo, NY 14215-1199
Shing Shing Yeh, Ph.D., M.D., Northport VA Medical Center
79 Middleville Road, Northport, NY 11768
Andrew W. Yen, M.D., VA Northern California Health Care System
10535 Hospital Way, Mather, CA 95655

© 2024 Triozzi JL et al. *JAMA Network Open*.
