# Supplementary material for: GLP1R Gene Expression and Kidney Disease Progression
Source: JAMA Netw Open. 2024 Oct 25;7(10):e2440286. doi: 10.1001/jamanetworkopen.2024.40286 (PMC11581634; doi:10.1001/jamanetworkopen.2024.40286)
Supplement: Supplement 3. — Data Sharing Statement [file jamanetwopen-e2440286-s003.pdf]

Data Sharing Statement

Triozzi. GLP1R Gene Expression and Kidney Disease Progression. *JAMA Netw Open*. Published October 25, 2024. doi:10.1001/jamanetworkopen.2024.40286

Data
Data available: No

Additional Information
Explanation for why data not available: All genetic instruments are provided in the supplemental materials. The GTEx Portal is available from https://gtexportal.org/. The VA MVP individual level data will not be shared through dbGAP; the Office of Research and Development of the Department of Veterans Affairs needs to be contacted for access requests for that data.
